# Dietary inflammatory index and its relationship with high-sensitivity C-reactive protein in Korean: data from the health examinee cohort

**DOI:** 10.3164/jcbn.17-22

**Published:** 2017-11-11

**Authors:** Woori Na, Misung Kim, Cheongmin Sohn

**Affiliations:** 1 Department of Food and Nutrition, Wonkwang University, 460 Iksan-daero, Iksan, Jeonbuk 54538, Korea

**Keywords:** dietary inflammatory index, hs-CRP, inflammation marker, Korean

## Abstract

Inflammation is associated with chronic disease. High-sensitivity C-reactive protein (hs-CRP) is a predictor of chronic disease. The dietary inflammatory index (DII) is used to determine the overall inflammatory potential of diet. A cross-sectional analysis of Health Examinee cohort data (2012–2014) from Korea was performed. Subjects were 40–79 years of age (8,332 males; 19,754 females). The DII was used to analyze the relationship between subject characteristics, nutrient intake, and the hs-CRP. Additionally, the relationship between DII and hs-CRP as a predictor of chronic disease was examined. The DII was divided into 4 quartile: Q1 = −7.21 to −1.88 (median: −3.020), Q2 = −1.87 to −0.02 (median: −0.410), Q3 = −0.01 to 1.87 (median = 0.870) and Q4 = 1.88 to 7.34 (median = 3.040). For each group, the carbohydrate/protein/fat intake ratio was Q1 = 66.7:16.6:19.2, Q2 = 67.2:15.6:18.7, Q3 = 67.3:15.1:18.4 and Q4 = 67.3:14.0:17.9. The odds of elevated hs-CRP were 1.241 times higher in participants with the most proinflammatory diets than those with the most anti-inflammatory diets [hs-CRP; odds ratio (95% confidence interval) for Q4 vs Q1: 1.241 (1.071, 1.438); *p* for trend = 0.002]. An association was found between a high DII and high levels of hs-CRP. The DII may be applied to measure the association between diet and chronic diseases.

## Introduction

Inflammation has been reported to influence pathogenesis and progress of diverse diseases.^(1)^ High sensitivity C-reactive protein (hs-CRP), nuclear factor κB (NF-κB), and interleukin-6 (IL-6) are known inflammatory indices. These indices have been reported to be closely associated with chronic diseases such as cardiovascular disease and cancer.^(2–6)^ In particular, the America Heart Association (AHA) and the Centers for Disease Control and Prevention (CDCP) used hs-CRP to propose a standard for measuring the health status of patients with cardiovascular disease.^(7)^ Recently, it has been proposed that these inflammatory indices may predict certain diseases.^(8)^ However, commonly used invasive method for measuring inflammation involves the collection and measurement of blood parameters.

The relationship between inflammation and dietary intake is being studied by examining the relationship of inflammation with fixed dietary patterns, newly developed dietary patterns, or certain nutrients. An adequate intake of vegetables and fruit has been reported to reduce CRP,^(9)^ people who follow the Mediterranean diet, recognized as a healthy dietary pattern, have reduced levels of hs-CRP and IL-6.^(10)^ The Mediterranean diet recommends a sufficient intake of whole grains, vegetables, fruit and fish, an adequate intake of alcohol and olive oil, and a reduced intake of red meat and high fat dairy products. In addition, some of the nutrients reported to reduce the risk of inflammation are complex carbohydrates,^(11)^ poly unsaturated fatty acid,^(12)^ dietary fiber,^(13)^ vitamin E^(14)^ and vitamin C.^(15)^ On the other hand, a Westernized dietary pattern that involves the intake of high fat red meat, high fat dairy products and refined grains has been reported to increase levels of hs-CRP.^(16)^

Shivappa *et al.*^(17)^ initially developed the dietary inflammatory index (DII). This index can be used to temporarily estimate inflammation levels through examination of dietary intake. The index was developed through the systematic examination of 36 nutrients and nine foods based on a literature review on cell experiments, animal experiments, and epidemiologic research. Compared to the more invasive method used for measuring inflammation, the DII may be used to non-invasively measure inflammation through dietary intake. Regarding progress in the field of research on the DII, research is being conducted to verify the validity of the relationship between DII and inflammatory index. Patients with metabolic syndrome with a high DII indicated a more significant increase in blood hs-CRP than patients with metabolic syndrome with a lower DII.^(18)^ Patients with asthma with a high DII had a more significant increase in IL-6.^(19)^

In the U.S., analysis of National Health and Nutrition Examination Survey III (NHANES III) data indicated that the risk of total mortality, cancer-related mortality, and gastrointestinal cancer mortality increased as the DII increased.^(20)^ In Spain, through data analysis of a high-risk cardiovascular disease group from PREDIMED (Prevención con Dieta Mediterránea), it was found that the risk of cerebrovascular disease increased as the DII increased.^(21)^ As a result of analyzing the Seguimiento Universidad de Navarra (SUN) cohort, it was found that the risk of cardiovascular disease increased as the DII increased.^(22)^ In France, as a result of analyzing the Supplementation en Vitamines et Mineraux Antioxydants (SU.VI.MAX) cohort, it was found that the risk of myocardial infarction increased as the DII increased.^(23)^ In Korea, as a result of analyzing the patient examination result data provided by the National Health Insurance Service, it was found that the risk of colorectal cancer increased as the DII increased.^(24)^ In addition, according to research conducted by Kim and Sohn,^(25)^ as a result of analyzing the DII among patients with metabolic syndrome through data from the Health Examinee Cohort, it was found that the risk of metabolic syndrome and the acceptance number indicating metabolic syndrome increased as the DII increased. As described, research is being conducted in diverse groups to evaluate the usefulness of the DII.

Although the DII is being researched in diverse nations, the majority of the research conducted in Korea is limited to certain diseases. A limited amount of research has incorporated healthy people without disease to analyze the relationship between the DII and inflammatory indices. In particular, even the research in Korea is limited to analyzing the risks of colorectal cancer and metabolic syndrome. Considering that the inflammatory index has been reported to be a useful index for predicting certain diseases, it is necessary to select healthy people without disease as the subjects for analyzing the correlation between the DII and the inflammatory index. Accordingly, the purpose of the present study is to select healthy Koreans as the subjects and to analyze the relationship between DII and hs-CRP and, thereby, propose the basic data for calculating a DII for Koreans.

## Materials and Methods

### Selecting research subjects

The epidemiological data from the Health Examinee Cohort used for the analysis conducted in the present study was collected from the Korean Genome and Epidemiology Study (KOGES) provided by the Department of Genomic Epidemiology at the Korea Centers for Disease Control and Prevention. KOGES is a study that collects and examines biological data, such as blood, urine, and genetic material, as well as epidemiological survey data. Subjects were selected from the general Korean population to construct a large-scale cohort. Since 2004, the Health Examinee cohort has been gathering new participants from 40 to 70 years of age mostly from examination/medical institutions nationwide. Approximately 20 medical institutions and 30,000 people nationwide participate in the survey annually. Surveys targeting people visiting hospitals for a physical examination were conducted in the regions of Seoul, Busan, Incheon, Daegu, Gwangju, Ulsan, Anyang of Gyeonggi, Goyang of Gyeonggi, Seongnam of Gyeonggi, Chuncheon of Gangwon, Cheonan of Chungnam, Hwasun of Jeonnam and Changwon of Gyeongnam.

In the present study, people over 40 years of age who participated in the Preventive Genome Health Examinee cohort included in KOGES from 2012 to 2014 were selected as subjects. Subject was excluded from that was had disease such as stroke, myocardial infarction, ischemic heart diseases and diabetes mellitus. As a result of conducting an open nutrition survey and biochemical examination survey, the data collected from 28,086 people consisting of 8,332 males and 19,754 females were analyzed, and research was conducted after acquiring the approval for the research ethics (WKIRB-201603-SB-016).

### Dietary intake and questionnaire data

KOGES examined the dietary intake of the participants based on a 24-h recall method. The primary interview survey was given to participants from the primary examination institution and the secondary telephone survey was given to those participants who completed the primary dietary intake survey. The primary interview survey data were used as the dietary intake survey data in the present study. An analysis was conducted through excluding data that indicated a daily intake of less than 500 kcal or more than 4,000 kcal.

From the data acquired from the Health Examinee Cohort, sociodemographic data collected through the survey was used for analyzing academic background and income level. The lifestyle survey data was used for analyzing smoking status and exercise status.

### Analyzing the dietary inflammatory index

The 24-h recall method data was used to calculate the z-value for analyzing the DII. The mean and standard deviation proposed by Shivappa *et al.*^(17)^ were used to calculate the z-value for analyzing 36 nutrients and nine foods. The mean and standard deviation of the nutrients and foods collected from the 11 nations (US, Australia, Bahrain, Denmark, India, Japan, New Zealand, Taiwan and Mexico), including Korea, were used for the analysis. To minimize the bias phenomenon of each nutrient and food listed in the DII, the percentile score was calculated. DII was calculated by multiplying the percentile score and sum of each food and nutrient. The DII was composed of for the following nutrients: vitamin B_12_, vitamin B_6_, beta-carotene, caffeine, carbohydrate, cholesterol, energy, eugenol, total fat, dietary fiber, folate, iron, magnesium, monounsaturated fatty acid, niacin, omega-3 fatty acid, omega-6 fatty acid, polyunsaturated fatty acid, protein, riboflavin, saturated fatty acid, selenium, thiamin, trans fatty acid, vitamin A, vitamin C, vitamin D, zinc, anthocyanidin, flavan-3-ol, flavanone, flavone, flavonol and isoflavone. The DII was proposed for the following foods: garlic, ginger, onion, saffron, turmeric, green tea, black tea, pepper, thyme/oregano and rosemary. The nutritional content data used in the present study were the functional ingredients table (Rural Development Administration), Computer Aided Nutritional Analysis (Journal of Nutrition and Health), and data provided by the U.S. Department of Agriculture. A low DII signified an anti-inflammatory diet, and a high DII signified an inflammatory diet.

### Statistical analysis

To analyze the relationship of the DII with the characteristics, nutrient intake, and inflammatory index of the subjects, the DII was divided into four levels for the analysis. For nutrient intake, the nutrient intake per 1,000 kcal was calculated. Since the hs-CRP was not normally distributed, it was log-transformed; thus, the results were log-transformed as well. In addition, hs-CRP was divided into two levels for analysis using 3 mg/L as the standard, which is the risk section proposed by the AHA/CDCP.

To analyze the characteristics and nutrient intake of the subjects according to the DII, an ANOVA and frequency analysis were conducted. Regarding the analysis of the subjects’ characteristics, to analyze the trend according to the DII, the *p* for trend was applied to the following continuous variables: age, body mass index (BMI), systolic pressure, diastolic pressure, fasting glucose, hs-CRP and white blood cell (WBC) count. In addition, to examine the relationship between DII and hs-CRP, the hs-CRP was used to perform logistic regression analysis; the odds ratio (OR) and confidence interval (CI) were calculated. All analyses were conducted using SPSS ver. 24.0.

## Results

### General characteristics according to the quartile of the dietary inflammatory index

The general characteristics of the subjects were analyzed by dividing the DII calculated in the present study into quartiles (Table 1). The DII was divided into: quartile 1 = −7.21 to −1.88 (median: −3.020), quartile 2 = −1.87 to −0.02 (median: −0.410), quartile 3 = −0.01 to 1.87 (median = 0.870) and quartile 4 = 1.88 to 7.34 (median = 3.040). For each quartile, the mean age was: quartile 1 = 53.38 years, quartile 2 = 52.67 years, quartile 3 = 52.20 years and quartile 4 = 51.64 years. DII increased as age decreased (*p*<0.001). For each quartile, the number of past smokers was: quartile 1 = 1,029 (27.2%), quartile 2 = 964 (25.5%), quartile 3 = 954 (25.3%) and quartile 4 = 831 (22.0%). The number of current smokers was quartile 1 = 644 (20.6%), quartile 2 = 740 (23.7%), quartile 3 = 797 (25.5%) and quartile 4 = 942 (30.2%). A statistically significant difference was indicated (*p*<0.001). The number of past smokers increased as the DII decreased, whereas the number of current smokers decreased as the DII decreased.

For each quartile, the mean of hs-CRP was: quartile 1 = 0.929, quartile 2 = 0.964, quartile 3 = 0.983 and quartile 4 = 1.024 (*p* for trend<0.000). The mean of WBC counts were quartile 1 = 5.522, quartile 2 = 5.580, quartile 3 = 5.616 and quartile 4 = 5.683 (*p* for trend<0.000). The hs-CRP and WBC counts significantly increased as the DII increased.

The hs-CRP was divided into two levels based on the level of risk. As a result of conducting a cross-tabulation analysis according to the quartiles of the DII, the number of subjects in the high hs-CRP level was: quartile 1 = 347 (21.9%), quartile 2 = 381 (24.1%), quartile 3 = 419 (26.5%) and quartile 4 = 435 (27.5%). The number of subjects in the low hs-CRP level, however, was: quartile 1 = 6,685 (25.2%), quartile 2 = 6,632 (25.0%), quartile 3 = 6,627 (25.0%) and quartile 4 = 6,560 (24.8%).

### Dietary intake according to the quartile of the dietary inflammatory index

The dietary intake of the subjects analyzed by dividing the DII into four quartile is shown in Table 2. For each quartile, the mean of energy intake was quartile 1 = 1,794.29 kcal, quartile 2 = 1,555.97 kcal, quartile 3 = 1,389.01 kcal and quartile 4 = 1,163.24 kcal. It was found that the energy intake decreased as the DII increased (*p*<0.001). In addition, although the protein/fat/carbohydrate intakes indicated similar results, when intakes were analyzed based on 1,000 kcal, the mean protein intake was: quartile 1 = 41.52 g, quartile 2 = 39.21 g, quartile 3 = 37.84 g and quartile 4 = 35.15 g (*p* = 0.000). The mean fat intake was: quartile 1 = 21.34 g, quartile 2 = 20.79 g, quartile 3 = 20.45 g and quartile 4 = 19.99 g (*p* = 0.000). The mean of carbohydrate intake was quartile 1 = 374.67 g, quartile 2 = 321.99 g, quartile 3 = 292.24 g and quartile 4 = 246.67 g (*p* = 0.000). For each group, the carbohydrate/protein/fat intake ratio was: quartile 1 = 66.7:16.6:19.2, quartile 2 = 67.2:15.6:18.7, quartile 3 = 67.3:15.1:18.4 and quartile 4 = 67.3:14.0:17.9. The protein and fat intake decreased as the DII increased, whereas the carbohydrate intake significantly increased as the DII increased.

### Risk of increase in hs-CRP according to the quartile of the dietary inflammatory index

A logistic regression analysis was conducted to analyze the risk of increase in inflammatory indices such as hs-CRP for each DII quartile. The results are shown in Table 3. The hs-CRP were divided into two levels, based on the standard proposed by the AHA/CDCP. As the DII increased, the increase in hs-CRP surpassing the presumed risk level was: quartile 2 = 1.107 (0.953–1.285), quartile 3 = 1.218 (1.052–1.410) and quartile 4 = 1.277 (1.105–1.477) (*p* for trend = 0.000).

## Discussion

The purpose of this study was to determine whether the DII is applicable to Korean populations. The data from the Health Examinee Cohort was used for selecting healthy Koreans as subjects for analyzing the validity of the DII by analyzing its relationship with hs-CRP.

In the present study, the DII was divided into quartile for the analysis, and the median for each level was −3.02, −0.41, 0.87 and 3.04, respectively. In research that analyzed the SUN cohort data from Spain, as a result of dividing the DII into quartiles, the median for each level was −3.18, −2.27, −1.40 and 0.30, respectively.^(22)^ In research that analyzed the Iowa Women’s Health Study (IWHS) cohort data from the U.S., as a result of dividing the DII into quartile, the median for each level was −3.14, −1.89, −0.39 and 1.85, respectively.^(26)^ In the present study, Koreans were selected as the subjects for analyzing the DII with the purpose of evaluating its validity. However, the results indicated that DII of the 4 quartile was relatively higher values compared to the results acquired in other nations. It may not be reasonable to use these results to conclude that healthy Koreans rely on a more inflammatory diet in comparison to healthy populations in other countries, since each nation may have different genetic components and lifestyles. In the future, it may be necessary to analyze the DII using data from another cohort study or other large-scale research studies. In addition, it seems that it would be necessary to further analyze the DII in Asian countries, such as Japan and China, in order to propose an adequately standardized DII suitable for Asian populations.

It was found that age significantly decreased and BMI significantly increased as the DII increased. In Shivappa *et al.*^(27)^ in which healthy Belgians were selected as subjects for examining the relationship between DII and inflammatorymarkers it was found that age and BMI indicated no significant difference. However, in the present study, it was found that age significantly decreased and that BMI increased as the DII increased. The reason for such differences is that in the research that analyzed the Askelpios study, healthy adults from 35 to 55 years of age were selected as the subjects. ^(27)^ However, in the present study, healthy adults and elderly adults from 40 to 70 years of age were selected as the subjects.

For smoking status, it was found that the number of current smokers increased and that the number of non-smokers decreased as the DII increased. These results were similar to those acquired from a research study that analyzed smoking status according to the DII.^(21,27)^ For exercise status, the number of people not exercising decreased, and the number of people exercising more than once a week increased, as the DII decreased.^(26)^ Similar results were indicated in research that analyzed the IWHS cohort data in the U.S. The number of people that rarely exercised decreased, and the number of people frequently exercising increased as the DII decreased.^(21)^ According to the results acquired in research that analyzed the PREDIMED cohort data in Spain, the MET (Metabolic Equivalent Task) value increased as the DII decreased.^(21)^ Similar research results of inflammatory and lifestyle indices signify that the people relying more on an anti-inflammatory diet tend to have a healthier lifestyle. It is difficult to say that DII levels directly influence lifestyle. However, it was confirmed that people relying more on an anti-inflammatory diet tend to achieve a lifestyle that enhances health.

In research that analyzed the Seasonal Variation of Blood Cholesterol Study (SEASONS) in the U.S., it was found that the hs-CRP increased as DII increased.^(28)^ Accordingly, these results were identical to results acquired in the present study. In addition, as a result of dichotomizing the hs-CRP at the level of 3 mg/L based on the standard provided by the AHA/CDCP, it was found that the number of subjects in the group with a high hs-CRP, increased as the DII increased. However, in research that analyzed the Askelpios study, there was no significant positive relationship between adherence to the pro-inflammatory diet (high DII score) and hs-CRP.^(27)^ Such results are contrary to those acquired in the present study. These discrepancies may be due to the difference in the age range of the subjects in the present study and those of the subjects in the study by Shivappa *et al.*,^(27)^ which analyzed the Askelpios study.

The present study found that the total energy intake decreased and the carbohydrate intake per 1,000 kcal increased as the DII increased, whereas fat and protein intake significantly decreased as the DII increased. These findings are identical to the results of several studies.^(20–22)^ It was found that total fat intake significantly decreased as the DII increased. However, in studies by Garcia-Arellano *et al.*^(21)^ and Ramallal *et al.*^(22)^ the fat intake increased. In particular, in the two research studies conducted in Spain, saturated fatty acid and monounsaturated fatty acid intake increased, whereas polyunsaturated fatty acid intake decreased.^(21,22)^ The results varied depending on the type of fat. In the future, it seems that it would be necessary to conduct an analysis, dividing the types of fat consumed by Koreans according to the DII.

In this study, as a result of conducting a logistic regression analysis to analyze the relationship between DII and hs-CRP, it was found that the risk of increase in hs-CRP increased as the DII increased. And, after adjusting the age, sex, BMI, total calorie intake, status of contraceptive consumption, education level, blood pressure status and physical activity, significant difference was indicated. According to Shivappa *et al.*,^(28)^ which analyzed SEASONS data, as a result of using the hs-CRP to conduct a regression analysis according to the DII, even after adjusting for age, race, blood cholesterol, marriage status, employment status, status of medication, status of alcohol, and functional food consumption, it was found that the risk of increase in hs-CRP increased as the DII increased.^(28)^ However, there was no positive association between the DII and hs-CRP in a Belgian cross-sectional study.^(27)^

We suggest several reasons for this inconsistent result regarding DII and hs-CRP. It may be due to the different races, dietary habits, and lifestyles in the study subjects. The previous validation study regarding the DII had been mostly performed in cancer patients in the US and Europe area. Therefore, validity studies of DII with several inflammatory mediated diseases are required in diversity populations. And, the overall inflammatory effect was initially applied to the development of the current DII. Since the DII was developed based on results acquired from research on diverse inflammatory indices (IL-1B, IL-4, IL-6, IL-10, tumor necrosis factor-α and hs-CRP), the results may be different from the results acquired from research that was focused on hs-CRP alone. Currently, hs-CRP is a known inflammatory indicator that can be used to judge the health status of individuals. The AHA/CDCP has proposed hs-CRP as a standard index for treating and monitoring patients with cardiovascular disease. In other words, because hs-CRP is an indicator for predicting pathogenesis of untreated cardiovascular disease, research is being conducted to review whether hs-CRP can be effectively used as an inflammatory index for people without disease. It may be necessary to adjust and supplement the DII so that it may be further developed and therefore provide a more accurate estimation than the current hs-CRP. However, it is important to conduct research that targets diverse subjects in order to examine the further elucidate the relationship between DII and hs-CRP among diverse populations.

The present study focused its analysis on the relationship between DII and hs-CRP. So, the relationship between DII and other inflammatory indices (IL-6 and NF-κB, which are known to relate to the pathogenesis of chronic diseases) was not additionally analyzed. However, the present study is significant in that it selected healthy people as the subjects, and analyzed the relationship of the DII with hs-CRP, which is known as the inflammatory index that corresponds to health status. However, the present study used a cross-sectional design, it was unable to examine the causal relationship between DII and the inflammatory index. Accordingly, further, prospective research is needed. In Korea, studies relating to the DII are lacking, and most of the research has focused on patients with diseases. Accordingly, the present study is significant in that it selected healthy Koreans as the subjects for analyzing the relationship between DII and hs-CRP. The results acquired in the present study may be used as the baseline data to conduct further research into DII, to provide more accurate estimates on levels of inflammation.

## Figures and Tables

**Table 1:** Characteristics, dietary inflammatory index

Characteristic	Quartile 1	Quartile 2	Quartile 3	Quartile 4	*p* value
	(*n* = 7,032)	(*n* = 7,013)	(*n* = 7,046)	(*n* = 6,995)	
Age (years)^†^	53.38^†^ ± 7.89	52.67 ± 7.965	52.2 ± 8.023	51.64 ± 8.039	<0.001
Sex					0.043
Men	2,150 (25.8^‡^)	2,105 (25.3)	2,087 (25)	1,990 (23.9)	
Women	4,882 (24.7)	4,908 (24.8)	4,959 (25.1)	5,005 (25.3)	
BMI (kg/m^2^)	23.46 ± 2.785	23.57 ± 2.856	23.67 ± 2.964	23.65 ± 3.03	<0.001
SBP (mmHg)	121.3 ± 14.783	121.24 ± 14.727	120.83 ± 14.704	121.05 ± 15.076	0.23
DBP (mmHg)	74.61 ± 9.727	74.76 ± 9.82	74.56 ± 9.616	75.01 ± 9.963	0.031
Fasting glucose (mg/dl)	91.99 ± 9.246	92 ± 9.225	91.87 ± 9.18	91.65 ± 9.305	0.089
Smoker					<0.001
Past, smoking	1,029 (27.2)	964 (25.5)	954 (25.3)	831 (22)	
Yes	644 (20.6)	740 (23.7)	797 (25.5)	942 (30.2)	
No	5,355 (25.3)	5,307 (25.1)	5,295 (25)	5,222 (24.7)	
Exercise practice (for a week)					<0.001
0	2,797 (20.7)	3,244 (24.1)	3,562 (26.4)	3,877 (28.8)	
1~2	951 (27.2)	885 (25.3)	883 (25.3)	773 (22.1)	
3~4	1,609 (29.3)	1,445 (26.3)	1,278 (23.3)	1,160 (21.1)	
5~6	941 (30.1)	819 (26.2)	738 (23.6)	632 (20.2)	
7	734 (29.5)	620 (24.9)	585 (23.5)	553 (22.2)	
Education					<0.001
Uneducated	94 (17.2)	109 (20)	142 (26)	201 (36.8)	
Elementary school	642 (23.1)	680 (24.5)	711 (25.6)	748 (26.9)	
Middle school	961 (24.4)	986 (25)	1,002 (25.4)	989 (25.1)	
High school	2,687 (24.8)	2,703 (24.9)	2,771 (25.5)	2,694 (24.8)	
University	2,238 (26.5)	2,144 (25.4)	2,051 (24.3)	1,997 (23.7)	
Graduate school	390 (27.7)	359 (25.5)	350 (24.9)	309 (21.9)	
Family income (10,000 won)					<0.001
less than 100	424 (20.9)	486 (23.9)	521 (25.6)	602 (29.6)	
100–less than 200	2,925 (25.4)	2,808 (24.3)	2,889 (25.1)	2,910 (25.2)	
300–less than 400	1,531 (24.5)	1,599 (25.6)	1,592 (25.5)	1,530 (24.5)	
400–less than 600	1,344 (26.5)	1,304 (25.8)	1,256 (24.8)	1,159 (22.9)	
Over 600	744 (27.7)	715 (26.6)	653 (24.3)	578 (21.5)	
Inflammatory markers					
hs-CRP (mg/L)	0.93 ± 1.151	0.96 ± 1.182	0.98 ± 1.188	1.02 ± 1.267	<0.001
log hs-CRP	–0.46 ± 0.794	−0.43 ± 0.812	−0.41 ± 0.813	−0.39 ± 0.832	<0.001
WBC blood (10^3^/µl)	5.52 ± 1.475	5.58 ± 1.475	5.62 ± 1.507	5.68 ± 1.543	<0.001
hs-CRP (>3 mg/L)					0.005
High	347 (21.9)	381 (24.1)	419 (26.5)	435 (27.5)	
Low	6,685 (25.2)	6,632 (25)	6,627 (25)	6,560 (24.8)	
DII score*	−3.21 ± 0.977	−0.92 ± 0.53	0.89 ± 0.542	3.24 ± 0.991	<0.001
Median	−3.02	−0.41	0.87	3.04	

^†^Mean ± SD, ^‡^*n* (%). *DII score range: Quartile 1 (−7.21 to −1.88); Quartile 2 (−1.87 to –0.02); Quartile 3 (−0.01 to 1.87); Quartile 4 (1.88 to 7.34).

**Table 2:** Dietary intake

Variable	Quartile 1	Quartile 2	Quartile 3	Quartile 4	*p* value
	(*n* = 7,032)	(*n* = 7,013)	(*n* = 7,046)	(*n* = 6,995)	
Energy (kcal)	1,794.29 ± 387.83^†^	1,555.97 ± 363.64	1,389.01 ± 360.37	1,163.24 ± 341.40	<0.001
Carbohydrate (g/1,000 kcal)	166.84 ± 20.45	168.07 ± 22.26	168.26 ± 24.22	169.88 ± 27.38	<0.001
Protein (g/1,000 kcal)	41.52 ± 7.37	39.21 ± 7.72	37.84 ± 8.34	35.15 ± 8.75	<0.001
Fat (g/1,000 kcal)	21.34 ± 7.06	20.79 ± 7.71	20.45 ± 8.32	19.99 ± 9.79	<0.001
Ca (mg/1,000 kcal)	374.67 ± 157.74	321.99 ± 143.76	292.24 ± 140.13	246.67 ± 135.59	<0.001
P (mg/1,000 kcal)	747.76 ± 128.44	692.94 ± 126.18	653.4 ± 126.01	588.25 ± 132.08	<0.001
Fe (mg/1,000 kcal)	10.77 ± 7.24	9.17 ± 3.12	8.17 ± 3.00	6.86 ± 2.87	<0.001
K (mg/1,000 kcal)	2,210.35 ± 543.61	1,927.74 ± 504.4	1,717.63 ± 466.4	1,411.61 ± 448.84	<0.001
Vitamin A (R.E/1,000 kcal)	787.54 ± 402.15	585.89 ± 337.01	463.52 ± 300.55	311.47 ± 229.29	<0.001
Vitamin B1 (mg/1,000 kcal)	0.86 ± 2.10	0.8 ± 2.19	0.77 ± 1.96	0.68 ± 2.09	<0.001
Vitamin B2 (mg/1,000 kcal)	0.79 ± 0.22	0.68 ± 0.21	0.63 ± 0.22	0.55 ± 0.27	<0.001
Niacin (mg/1,000 kcal)	10.55 ± 2.64	9.6 ± 2.56	9.03 ± 2.68	7.96 ± 2.49	<0.001
Vitamin C (mg/1,000 kcal)	88.68 ± 41.09	68.67 ± 35.7	55.96 ± 32.93	38.33 ± 26.36	<0.001
Vitamin E (mg/1,000 kcal)	7.24 ± 2.17	6.1 ± 2.06	5.41 ± 2.01	4.4 ± 2.05	<0.001
CHO:PRO:FAT	66.73:16.60:19.20	67.23:15.68:18.71	67.30:15.13:18.40	67.95:14.06:17.99	
CHO (%)	66.73 ± 8.18	67.23 ± 8.90	67.3 ± 9.69	67.95 ± 10.95	<0.001
PRO (%)	16.6 ± 2.94	15.68 ± 3.08	15.13 ± 3.33	14.06 ± 3.5	<0.001
FAT (%)	19.2 ± 6.35	18.71 ± 6.93	18.4 ± 7.49	17.99 ± 8.81	<0.001

^†^Mean ± SD.

**Table 3:** Associations between the dietary inflammatory index and hs-CRP

Variable	Quartile 1	Quartile 2	Quartile 3	Quartile 4	*p* for trend
		OR (95% CI)	OR (95% CI)	OR (95% CI)	
hs-CRP (>3 mg/L or ≤3 mg/L)					
Crude	1 (reference)	1.107 (0.953 to 1.285)^†^	1.218 (1.052 to 1.410)	1.277 (1.105 to 1.477)	<0.001
Adjusted 1^‡^	1	1.106 (0.952 to 1.286)	1.205 (1.040 to 1.397)	1.287 (1.111 to 1.490)	<0.001
Adjusted 2^§^	1	1.084 (0.932 to 1.260)	1.176 (1.014 to 1.364)	1.241 (1.071 to 1.438)	0.002

^†^OR (95% CI); Odds ratios (95% confidence interval). ^‡^Adjusted 1: age, sex, BMI. ^§^Adjusted 2: age, sex, BMI, smoking status, education level, blood pressure status, total calorie intake, use of oral contraceptive and physical activity.

## Abbreviations

DII: dietary inflammatory index
hs-CRP: high-sensitivity C-reactive protein
IL: interluekin
KOGES: Korean Genome and Epidemiology Study
MET: Metabolic Equivalent Task
NF-κB: nuclear factor-κB
WBC: white blood cell

## References

[1] Feingold KR, Grunfeld C (1992). Role of cytokines in inducing hyperlipidemia. Diabetes.

[2] Mohamed-Ali V, Goodrick S, Rawesh A (1997). Subcutaneous adipose tissue releases interleukin-6, but not tumor necrosis factor-α, *in vivo* 1. J Clin Endocrinol Metab.

[3] Tracy RP, Lemaitre RN, Psaty BM (1997). Relationship of C-reactive protein to risk of cardiovascular disease in the elderly. Arterioscler Thromb Vasc Biol.

[4] Ridker PM (2001). High-sensitivity C-reactive protein: potential adjunct for global risk assessment in the primary prevention of cardiovascular disease. Circulation.

[5] Ridker PM (2003). Clinical application of C-reactive protein for cardiovascular disease detection and prevention. Circulation.

[6] Gabay C, Kushner I (1999). Acute-phase proteins and other systemic responses to inflammation. N Engl J Med.

[7] Pearson TA, Mensah GA, Alexander RW (2003). Markers of inflammation and cardiovascular disease: application to clinical and public health practice: a statement for healthcare professionals from the Centers for Disease Control and Prevention and the American Heart Association. Circulation.

[8] Gotsman I, Stabholz A, Planer D (2008). Serum cytokine tumor necrosis factor-alpha and interleukin-6 associated with the severity of coronary artery disease: indicators of an active inflammatory burden?. Isr Med Assoc J.

[9] Esmaillzadeh A, Kimiagar M, Mehrabi Y, Azadbakht L, Hu FB, Willett WC (2006). Fruit and vegetable intakes, C-reactive protein, and the metabolic syndrome. Am J Clin Nutr.

[10] Esposito K, Marfella R, Ciotola M (2004). Effect of a mediterranean-style diet on endothelial dysfunction and markers of vascular inflammation in the metabolic syndrome: a randomized trial. JAMA.

[11] Kitabchi AE, McDaniel KA, Wan JY (2013). Effects of high-protein versus high-carbohydrate diets on markers of β-cell function, oxidative stress, lipid peroxidation, proinflammatory cytokines, and adipokines in obese, premenopausal women without diabetes. Diabetes Care.

[12] Ferrucci L, Cherubini A, Bandinelli S (2006). Relationship of plasma polyunsaturated fatty acids to circulating inflammatory markers. J Clin Endocrinol Metab.

[13] Ma Y, Griffith JA, Chasan-Taber L (2006). Association between dietary fiber and serum C-reactive protein. Am J Clin Nutr.

[14] Bertran N, Camps J, Fernandez-Ballart J (2005). Diet and lifestyle are associated with serum C-reactive protein concentrations in a population-based study. J Lab Clin Med.

[15] Wannamethee SG, Lowe GD, Rumley A, Bruckdorfer KR, Whincup PH (2006). Associations of vitamin C status, fruit and vegetable intakes, and markers of inflammation and hemostasis. Am J Clin Nutr.

[16] Johansson-Persson A, Ulmius M, Cloetens L, Karhu T, Herzig KH, Önning G (2014). A high intake of dietary fiber influences C-reactive protein and fibrinogen, but not glucose and lipid metabolism, in mildly hypercholesterolemic subjects. Eur J Nutr.

[17] Shivappa N, Steck SE, Hurley TG, Hussey JR, Hébert JR (2014). Designing and developing a literature-derived, population-based dietary inflammatory index. Public Health Nutr.

[18] Wirth M, Burch J, Shivappa N (2014). Association of a dietary inflammatory index with inflammatory indices and the metabolic syndrome among police officers. J Occup Env Med.

[19] Wood LG, Shivappa N, Berthon BS, Gibson PG, Hebert JR (2015). Dietary inflammatory index is related to asthma risk, lung function and systemic inflammation in asthma. Clin Exp Allergy.

[20] Shivappa N, Steck SE, Hussey JR, Ma Y, Hebert JR (2017). Inflammatory potential of diet and all-cause, cardiovascular, and cancer mortality in National Health and Nutrition Examination Survey III Study. Eur J Nutr.

[21] Garcia-Arellano A, Ramallal R, Ruiz-Canela M (2015). Dietary inflammatory index and incidence of cardiovascular disease in the PREDIMED study. Nutrients.

[22] Ramallal R, Toledo E, Martínez-González MA (2015). Dietary inflammatory index and incidence of cardiovascular disease in the SUN cohort. PLoS One.

[23] Neufcourt L, Assmann KE, Fezeu LK (2016). Prospective association between the dietary inflammatory index and cardiovascular diseases in the SUpplémentation en Vitamines et Minéraux AntioXydants (SU.VI.MAX) cohort. J Am Heart Assoc.

[24] Cho Y, Lee J, Oh JH, Shin A, Kim J (2016). Dietary inflammatory index and risk of colorectal cancer: a case-control study in Korea. Nutrients.

[25] Kim M, Sohn C (2016). Analysis of dietary inflammatory index of metabolic syndrome in Korean: data from the health examinee cohort (2012–2014).. Korean J Hum Ecol.

[26] Shivappa N, Blair CK, Prizment AE, Jacobs DR, Steck SE, Hébert JR (2016). Association between inflammatory potential of diet and mortality in the Iowa Women’s Health study. Eur J Nutr.

[27] Shivappa N, Hébert JR, Rietzschel ER (2015). Associations between dietary inflammatory index and inflammatory markers in the Asklepios Study. Br J Nutr.

[28] Shivappa N, Steck SE, Hurley TG (2014). A population-based dietary inflammatory index predicts levels of C-reactive protein in the Seasonal Variation of Blood Cholesterol Study (SEASONS).. Public Health Nutr.

